# When awareness is not enough: online fraud susceptibility, threat awareness, anti-fraud self-efficacy and online scam prevention behavior among Chinese university students

**DOI:** 10.3389/fpsyg.2026.1857855

**Published:** 2026-07-07

**Authors:** Liwen Chen, Hanqiang Li, Yan Piaw Chua, Jian Chen

**Affiliations:** 1 College of Tourism and Historical Culture, Chizhou University, Chizhou, China; 2 Faculty of Social Sciences and Liberal Arts, UCSI University, Cheras, Malaysia

**Keywords:** anti-fraud self-efficacy, Chinese university students, online fraud susceptibility, online scam prevention behavior, protection motivation theory, threat awareness

## Abstract

**Objective:**

As university students’ learning, social interaction, and payment activities increasingly move online, online scam prevention has become an important issue in digital safety governance in Chinese higher education. Drawing on Protection Motivation Theory, this study examined the relationships among online fraud susceptibility, threat awareness, anti-fraud self-efficacy, and online scam prevention behavior, and further explored latent group differences in online fraud susceptibility.

**Methods:**

Data were collected from 765 university students in China. Structural equation modeling was used to test the relationships among the focal variables. Latent profile analysis, combined with the Bolck–Croon–Hagenaars method, was then conducted to compare differences in threat awareness, anti-fraud self-efficacy, and online scam prevention behavior across latent profiles.

**Results:**

Online fraud susceptibility was significantly and negatively associated with threat awareness, anti-fraud self-efficacy, and online scam prevention behavior. Both threat awareness and anti-fraud self-efficacy were significantly and positively associated with online scam prevention behavior, whereas the path from threat awareness to anti-fraud self-efficacy was not significant. The mediation results suggest that threat awareness and anti-fraud self-efficacy were statistically supported as two indirect parallel pathways rather than as a stable sequential process. Latent profile analysis identified three groups: low-susceptibility, moderate-susceptibility, and high-susceptibility. The low-susceptibility group showed better overall performance in threat awareness, anti-fraud self-efficacy, and online scam prevention behavior than the other two groups, whereas the difference between the moderate-susceptibility and high-susceptibility groups was mainly reflected in anti-fraud self-efficacy.

**Conclusion:**

Differences in online scam prevention behavior among Chinese university students are associated not only with general risk awareness, but also with susceptibility structure, efficacy beliefs, and latent group heterogeneity. The findings suggest that university anti-fraud education should place greater emphasis on strengthening anti-fraud self-efficacy and providing differentiated support for students with different levels of susceptibility.

## 1 Introduction

The rapid spread of mobile internet, social media, instant messaging, and online payment has gradually shifted a growing share of everyday activities into digital spaces, making online scams a common risk in contemporary online life (Norris et al., 2019; Vishwanath et al., 2011). From the perspective of Routine Activity Theory, the expansion of online activity increases individuals’ opportunities to encounter potential offenders, thereby raising the likelihood of becoming scam targets (Cohen and Felson, 1979; Pratt et al., 2010; Reyns, 2015). University students are especially relevant in this regard because their study, social interaction, and consumption are deeply embedded in online platforms, making them frequent recipients of scam-related information and an important at-risk group in the online scam context (Lin et al., 2025; Ministry of Public Security of the People’s Republic of China, 2024). In the Chinese context, this group is also important because students are moving toward greater financial autonomy while still developing independent risk judgment and fraud-response experience, and universities provide an organized setting for anti-fraud education and behavioral intervention. Yet exposure alone does not determine whether individuals ultimately fall victim to scams. Routine online activity may increase encounters with suspicious information, but the consequences of such encounters also depend on risk recognition, prudent judgment, and response control in specific situations (Lin et al., 2025; Partin et al., 2022; van Wilsem, 2013).

Recent studies have therefore moved from single-factor explanations to multidimensional perspectives, suggesting that online fraud susceptibility is shaped by information-processing style, risk preference, anti-fraud knowledge, social influence, and related factors (Ren and Luo, 2022; Sun et al., 2024; Wang et al., 2025). From this perspective, online fraud susceptibility is better understood not as a one-dimensional trait, but as a broader psychological vulnerability involving cognitive, motivational, and behavioral tendencies. Even so, existing research has focused mainly on construct identification and scale development, while still offering limited explanation of how online fraud susceptibility relates to risk-related cognition and online scam prevention behavior (Ren and Luo, 2022; Sun et al., 2024; Wang et al., 2025). This gap is important because online scam prevention requires more than recognizing risk in general. Existing evidence indicates that phishing-related training can improve users’ ability to recognize risks, yet its effect on sustained behavioral change remains limited, and some trained users still fail to adopt effective preventive behavior in real-world situations (Marshall et al., 2024). In online scam contexts, individuals often need to complete risk recognition, information verification, and behavioral decision-making within a very limited time frame (Vishwanath et al., 2011; Musuva et al., 2019). Thus, knowledge transmission or general risk reminders alone may be insufficient to explain why university students differ in online scam prevention behavior.

For universities, this issue is not only about identifying victimization risk, but also about whether anti-fraud education should focus primarily on risk reminders, capability training, or differentiated support. Without a more fine-grained understanding of the factors associated with online scam prevention behavior among Chinese university students and of how these factors are structurally related, anti-fraud education in universities is likely to remain at the level of generalized publicity and may struggle to respond to actual differences across student groups in risk cognition, efficacy beliefs, and behavioral performance. Given that telecom and online fraud is the term commonly used in China’s institutional and governance context, and that the Chinese scales drawn upon in this study also adopt this expression, this article uses telecom and online fraud when defining the research context. However, to maintain consistency with the terms online scam and online fraud used in the international literature, and for the sake of concise expression, online scam is used hereafter unless otherwise specified.

Against this background, the present study shifts attention from explaining victimization risk alone to examining the relational structure of online scam prevention behavior among Chinese university students. Specifically, it addresses three questions: (1) What relationship exists between online fraud susceptibility and online scam prevention behavior? (2) What roles do threat awareness and anti-fraud self-efficacy play in the relational structure linking online fraud susceptibility and online scam prevention behavior? (3) Do Chinese university students display distinct latent profiles of online fraud susceptibility, and do these profiles differ systematically in threat awareness, anti-fraud self-efficacy, and online scam prevention behavior?

To address these questions, this study adopts Protection Motivation Theory as its main explanatory framework and examines the relationships among online fraud susceptibility, threat awareness, anti-fraud self-efficacy, and online scam prevention behavior among Chinese university students from both variable-centered and person-centered perspectives.

## 2 Literature review and hypotheses development

### 2.1 Online scam prevention behavior from the perspective of protection motivation theory

Exposure-based perspectives, such as Routine Activity Theory, help explain why university students may encounter online scam risks more frequently (Cohen and Felson, 1979; Reyns, 2015). However, these perspectives mainly address exposure to risk rather than differences in recognition, judgment, and preventive behavior after exposure. The present study therefore focuses less on exposure itself and more on why individuals facing similar online scam situations may show different preventive responses. Protection Motivation Theory provides a more direct and analytically suitable framework. Initially developed to explain protective responses under threat appeal conditions, Protection Motivation Theory was later refined through the incorporation of self-efficacy and gradually developed into a framework centered on threat appraisal and coping appraisal (Rogers, 1975; Maddux and Rogers, 1983). In research on information security and online safety, this theory has been widely used to explain variation in protective online behavior (Ifinedo, 2012; Liang and Xue, 2010; Tsai et al., 2016). In this study, threat awareness is used as the context-specific operational expression of perceived threat in PMT. It refers to students’ appraisal of the existence, seriousness, and personal relevance of online scam threats, whereas anti-fraud self-efficacy refers to their confidence in identifying, verifying, and responding to such threats.

Accordingly, this study adopts Protection Motivation Theory as the main framework for explaining variation in online scam prevention behavior among Chinese university students, while risk exposure is not specified as a direct path in the structural model.

### 2.2 Online fraud susceptibility and threat awareness

Online fraud susceptibility refers to the tendency to make erroneous judgments and compliant responses more readily when confronted with fraudulent online information (Norris et al., 2019). Existing research suggests that this tendency is jointly shaped by multiple factors, including information-processing style, risk preference, anti-fraud knowledge, self-control, and social influence (Sun et al., 2024; Wang et al., 2025). From an information-processing perspective, scam messages often use time pressure, situational inducement, and cue manipulation to reduce careful processing and increase reliance on heuristic judgment (Musuva et al., 2019; Chen et al., 2023). When cue recognition is weak and careful processing is constrained, individuals may form weaker risk judgments. Within the PMT framework, individuals with higher levels of online fraud susceptibility may therefore be more likely to underestimate risk or overlook critical warning signals, and consequently to exhibit lower levels of threat awareness.

The OFSS was originally developed as a five-factor measure, including monetary motivation, heuristic processing, financial risk preference, anti-fraud knowledge, and susceptibility to social influence. These dimensions capture related but distinguishable susceptibility mechanisms grounded in persuasion, information-processing, and decision-making perspectives (Wang et al., 2025). Accordingly, online fraud susceptibility in the present study was conceptualized as a multidimensional vulnerability structure rather than as a narrow single-facet trait. On this basis, the present study proposes the following hypotheses:

*H1*: Online fraud susceptibility is significantly and negatively associated with threat awareness.

### 2.3 Threat awareness and anti-fraud self-efficacy

Protection Motivation Theory distinguishes threat appraisal from coping appraisal. Threat appraisal concerns individuals’ evaluation of the seriousness and relevance of a potential risk, whereas coping appraisal concerns whether an effective response is available and whether individuals believe they are capable of carrying it out (Maddux and Rogers, 1983). From this perspective, threat awareness may be positively related to anti-fraud self-efficacy. In the online scam context, when individuals recognize more clearly the reality, seriousness, and personal relevance of online scam threats, they may be more likely to mobilize existing knowledge and prior experience and, in turn, form more positive judgments about their ability to identify and respond to scams. However, threat awareness concerns perceived risk, whereas anti-fraud self-efficacy concerns perceived coping capability. The two constructs are therefore related but conceptually distinct (Liang and Xue, 2010; Tsai et al., 2016; Sommestad et al., 2015). Accordingly, although a positive association may be expected, this does not necessarily mean that the relationship will take the form of a stable sequential pattern in every specific context. In other words, recognizing online scam risks may increase vigilance, but it may not automatically generate confidence in performing concrete anti-fraud actions.

This distinction is also consistent with adjacent research on digital risk behavior. For example, Paciello et al. (2023) showed that self-efficacy in dealing with misinformation involves both fact-checking and inhibitory self-regulation. Although misinformation and online scams differ, both require individuals to evaluate uncertain cues and regulate responses under pressure. Therefore, anti-fraud self-efficacy may be associated with threat awareness, but it should not be treated merely as a direct consequence of threat awareness.

Online fraud susceptibility may also be linked to coping appraisal more directly. Self-efficacy theory suggests that efficacy beliefs are shaped jointly by individuals’ cognitive style, prior experience, and self-regulatory capacity (Bandura, 1997; Pajares, 1996). In the online scam context, students who are more easily influenced by situational cues, lack anti-fraud knowledge, or have weaker self-regulation may feel less capable of dealing effectively with suspicious information and risky situations. Thus, anti-fraud self-efficacy may be shaped both by students’ awareness of scam-related threats and by their own susceptibility-related characteristics. On this basis, the study proposes the following hypotheses:

*H2*: Threat awareness is significantly and positively associated with anti-fraud self-efficacy.

*H3*: Online fraud susceptibility is significantly and negatively associated with anti-fraud self-efficacy.

### 2.4 Anti-fraud self-efficacy and online scam prevention behavior

Within Protection Motivation Theory, coping appraisal is closely tied to behavioral enactment, and self-efficacy is one of its central components. A substantial body of information security research has shown that self-efficacy is positively associated with security-related and protective behavior (Rhee et al., 2009; Ifinedo, 2012; Liang and Xue, 2010). In the online scam context, students who believe that they can identify suspicious information, judge its credibility, and respond effectively are more likely to engage in online scam prevention behavior.

Threat awareness may also be directly related to such behavior. Protection Motivation Theory suggests that individuals are more likely to engage in avoidance or protective action when they perceive a threat as real and consequential (Sommestad et al., 2015). In the context of online scams, if individuals recognize the seriousness of the threat and perceive it as personally relevant, they may be more likely to adopt preventive responses. On this basis, the following hypotheses are proposed:

*H4*: Anti-fraud self-efficacy is significantly and positively associated with online scam prevention behavior.

*H5*: Threat awareness is significantly and positively associated with online scam prevention behavior.

### 2.5 Online fraud susceptibility and online scam prevention behavior

The association between online fraud susceptibility and online scam prevention behavior may not be limited to indirect pathways through psychological appraisal. It may also exist at a more direct level. Prior research indicates that low self-control, stronger risk preference, and insufficient knowledge not only increase the likelihood of victimization but also undermine prudent judgment and behavioral control in risky situations (Norris et al., 2019; Holtfreter et al., 2008; van Wilsem, 2013). From this perspective, individuals with higher levels of online fraud susceptibility may be less likely to engage in careful verification, delay risky responses, or adopt other protective actions when confronted with suspicious online information. Accordingly, higher online fraud susceptibility is expected to be associated with lower levels of online scam prevention behavior.

*H6*: Online fraud susceptibility is significantly and negatively associated with online scam prevention behavior.

### 2.6 Research model

**Figure 1:**
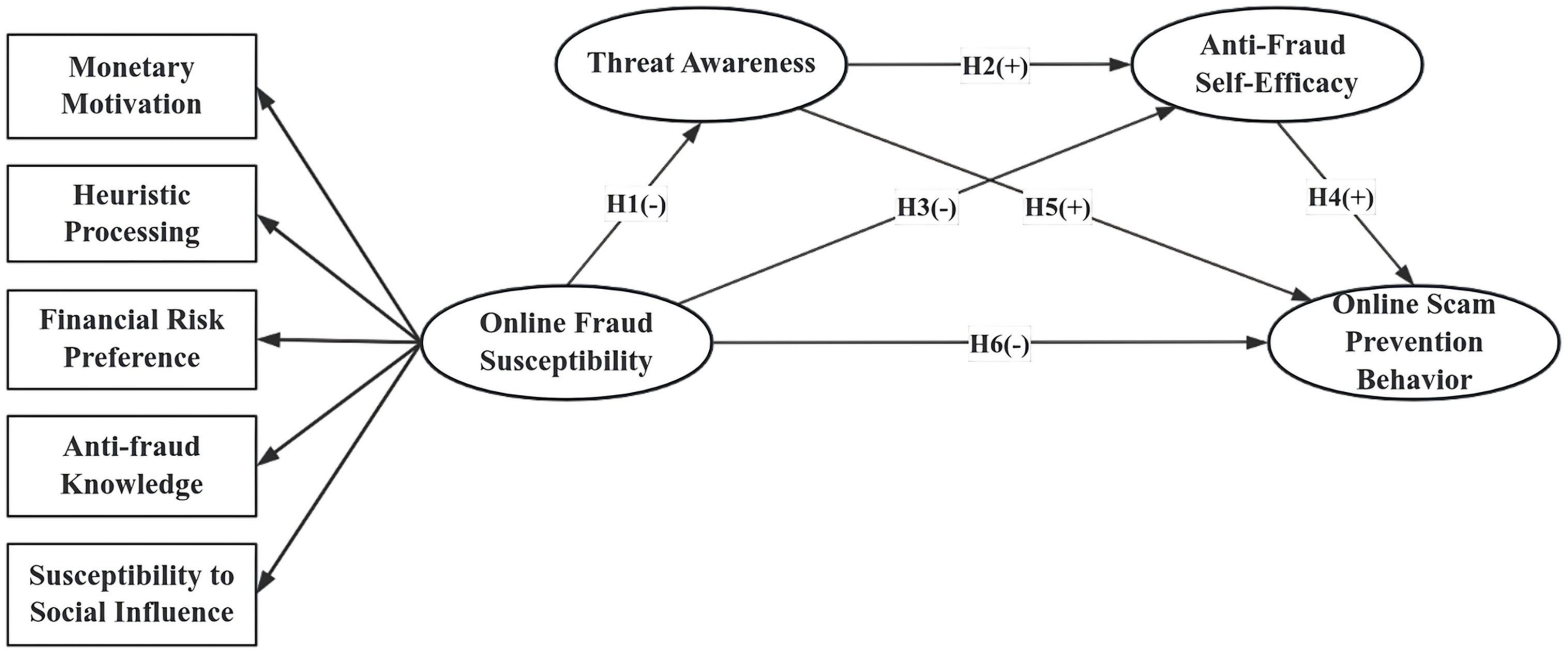
Proposed conceptual model of the study.

Based on the preceding theoretical arguments, this study develops a structural model in which online fraud susceptibility is specified as the focal explanatory construct and online scam prevention behavior as the focal behavioral construct. Within this model, threat awareness and anti-fraud self-efficacy correspond primarily to the threat appraisal and coping appraisal components of Protection Motivation Theory, respectively. The path from threat awareness to anti-fraud self-efficacy is retained because threat appraisal may precede coping appraisal in the classical PMT framework, but this path is treated as an empirical question rather than a necessary assumption. The proposed relationships among the focal variables are shown in Figure 1.

## 3 Methodology

### 3.1 Research design and data collection

This study adopted a cross-sectional questionnaire design and combined variable-centered and person-centered approaches in the empirical analysis. Specifically, structural equation modeling was first used to examine the relationships among online fraud susceptibility, threat awareness, anti-fraud self-efficacy, and online scam prevention behavior, together with the pattern of indirect effects. Building on this analysis, latent profile analysis was further employed to identify latent heterogeneity across the five dimensions of online fraud susceptibility among university students, and the Bolck–Croon–Hagenaars method was used to compare differences in threat awareness, anti-fraud self-efficacy, and online scam prevention behavior across latent profiles. This analytical strategy makes it possible to examine online scam prevention behavior both in terms of overall intervariable relationships and in terms of latent subgroup differentiation, thereby providing an empirical basis for identifying priorities and differentiated support in university anti-fraud education.

The target population consisted of students enrolled in Chinese higher education institutions. Data were collected through an online questionnaire distributed on the Wenjuanxing platform. The survey was disseminated using a combination of convenience sampling and snowball sampling, mainly through university class groups, student social media platforms, and peer networks. Both convenience sampling and snowball sampling are non-probability sampling strategies and are commonly used when a complete sampling frame is difficult to obtain and when accessibility and coverage are prioritized (Biernacki and Waldorf, 1981; Etikan et al., 2016). Eligible participants were current students at Chinese universities or colleges. In practice, participants were mainly recruited through student networks, class groups, and peer contacts in higher education institutions located in Anhui Province, China. Because the questionnaire was anonymous and did not collect university names, university locations, or institution identifiers, the exact number and proportion of participants from each institution could not be calculated. Therefore, the sample should be understood as a non-probability sample recruited mainly through Anhui-based higher education networks, rather than as a nationally representative sample of Chinese university students.

The study followed the principles of anonymity, voluntariness, and informed consent. On the first page of the questionnaire, participants were informed of the purpose of the study, the voluntary nature of participation, the anonymous handling of responses, and the fact that the data would be used solely for academic research. Participants could proceed to the formal survey only after reading and agreeing to these statements. No directly identifiable personal information was collected at any stage of the study. A total of 954 questionnaires were returned. After data collection, the raw responses were screened according to several predefined criteria. Responses were excluded if participants did not provide informed consent, failed to complete the questionnaire, showed missing values on focal variables, completed the questionnaire in an unrealistically short time relative to the length of the instrument, or displayed obvious invariant response patterns across most scale items. No statistical imputation was conducted because invalid cases were removed before the main analyses. After applying these screening criteria, 765 valid cases were retained from the 954 returned questionnaires, yielding an effective response rate of 80.19%. The demographic characteristics of the final sample are presented in Table 1.

**Table 1:** Demographic characteristics of the sample (*N* = 765).

Variable	Category	*n*	%
Gender	Male	304	39.74
	Female	461	60.26
Grade	First year	350	45.75
	Second year	155	20.26
	Third year	176	23.01
	Fourth year	84	10.98
Major category	Science and engineering	302	39.48
	Economics and management	188	24.58
	Humanities and history	188	24.58
	Arts and sports	46	6.01
	Other	41	5.36
Monthly living expenses	Below RMB 1,000	61	7.97
	RMB 1,000–2,000	639	83.53
	RMB 2,000–3,000	58	7.58
	Above RMB 3,000	7	0.92
Daily internet use	Less than 1 h	34	4.44
	1–3 h	177	23.14
	3–6 h	332	43.40
	More than 6 h	222	29.02

### 3.2 Measures

All items of variables were rated on a five-point Likert scale (1 = strongly disagree, 5 = strongly agree). The measures were primarily derived from established instruments or mature theoretical frameworks and were contextually adapted where necessary to fit the online scam context among Chinese university students. To ensure the accuracy of item wording and the usability of the measures, the scales that involved more substantial modification were subjected to bilingual translation, bilingual comparison, and expert review before the formal survey. The expert review involved three scholars from the fields of psychology and education, who examined the adapted items in terms of conceptual relevance, wording clarity, and contextual appropriateness for Chinese university students. Several expressions were subsequently revised in light of the feedback received (Beaton et al., 2000; Sousa and Rojjanasrirat, 2011). After data collection, measurement quality was further evaluated through confirmatory factor analysis, composite reliability, and average variance extracted, and underperforming items were handled according to the commonly used criterion that standardized factor loadings should exceed 0.50 (Boateng et al., 2018).

#### 3.2.1 Online fraud susceptibility

Online fraud susceptibility was measured using the Online Fraud Susceptibility Scale developed by Wang et al. (2025). The scale contains five dimensions: monetary motivation, heuristic processing, financial risk preference, anti-fraud knowledge, and susceptibility to social influence, with 25 items in total. The heuristic processing and anti-fraud knowledge dimensions were reverse coded in accordance with the original scale design. Because the present study focused on Chinese university students, whereas some items in the original instrument referred to income, consumption, and investment in ways more typical of general adult experience, only limited wording adjustments were made to a small number of items in order to improve comprehensibility and contextual fit for a student sample. These revisions did not alter the structure, dimensional configuration, or core meaning of the original instrument (Beaton et al., 2000; Sousa and Rojjanasrirat, 2011).

The original scoring logic of the scale was retained, such that all five dimensions were interpreted in the direction of victimization susceptibility. Accordingly, higher scores indicate stronger susceptibility in the corresponding aspect. After reverse coding, higher scores on the anti-fraud knowledge dimension indicate a lower level of anti-fraud knowledge, whereas higher scores on the heuristic processing dimension indicate a stronger tendency to rely on intuitive judgment rather than careful analysis. This is consistent with the overall logic of the original scale (Wang et al., 2025). Representative items include “I am not satisfied with my current financial situation” for monetary motivation, “When making decisions, I take time to weigh the pros and cons carefully and analyze risks and benefits” for heuristic processing, “I would spend a relatively large amount of my living expenses on high-risk investments or speculative opportunities” for financial risk preference, “I understand common telecom and online fraud tactics” for anti-fraud knowledge, and “When others pressure my decisions, I usually compromise” for susceptibility to social influence.

After formal data collection, the measurement performance of the scale was evaluated through confirmatory factor analysis. Following the commonly accepted criterion that standardized factor loadings should exceed 0.50 (Hair et al., 2019), one item from the monetary motivation dimension was removed. After revision, standardized factor loadings ranged from 0.617 to 0.958, composite reliability values ranged from 0.847 to 0.947, and average variance extracted values ranged from 0.585 to 0.786. Cronbach’s alpha coefficients for the five first-order dimensions were 0.842 for monetary motivation, 0.902 for heuristic processing, 0.936 for financial risk preference, 0.901 for anti-fraud knowledge, and 0.881 for susceptibility to social influence, all of which met recommended standards (Nunnally and Bernstein, 1994). These results indicate that the scale showed good internal consistency and convergent validity in the present sample.

#### 3.2.2 Threat awareness

Threat awareness was defined in this study as students’ context-specific perceived threat regarding online scams. In line with Protection Motivation Theory, it captures students’ judgment of the seriousness of online scam risks and their perceived likelihood of being exposed to or affected by such risks. Thus, threat awareness is not treated as a construct separate from perceived threat in PMT, but as its operational expression in the online scam context.

At the measurement level, this study drew on the wording used by Intarakamhang et al. (2025) in their measure of online risk or threat awareness and made moderate contextual adjustments for the online scam context. The purpose of these revisions was to ensure that the items more closely captured Chinese university students’ judgments about the reality of online scam risk, the seriousness of its potential consequences, and its personal relevance. Representative items include “I think online scams can have serious consequences for an individual’s life and property” and “I think being careless online makes a person more likely to encounter online scams,” which, respectively, reflect judgments of threat severity and likelihood of victimization.

The items were designed to capture the perceived reality of online scam risk, the seriousness of its potential consequences, and the likelihood of becoming a victim, while avoiding direct measurement of coping ability, behavioral frequency, or specific preventive skills. The scale consisted of six items. Standardized factor loadings ranged from 0.681 to 0.918, composite reliability was 0.935, average variance extracted was 0.706, and Cronbach’s alpha was 0.931, all of which met recommended standards (Hair et al., 2019). These results indicate good internal consistency and convergent validity.

#### 3.2.3 Anti-fraud self-efficacy

The measure of anti-fraud self-efficacy was informed by the task-based logic of the Internet Self-Efficacy Scale developed by Eastin and LaRose (2000). The core of this measurement approach lies not in specific internet-related technical terminology, but in assessing individuals’ subjective beliefs about their ability to complete relevant online tasks. Because the original scale was designed for general internet-use contexts, whereas the present study focused on the more specific and risk-oriented domain of online scam prevention, anti-fraud self-efficacy was operationalized in line with the theoretical logic of domain-specific self-efficacy (Bandura, 2006).

The items were designed around key requirements involved in preventing online scams, including identifying suspicious information, judging information credibility, verifying suspicious requests, protecting personal information, handling potential scam situations, and seeking help or reporting. Representative items include “I feel confident judging whether online information is trustworthy” and “Before responding to online information, I feel confident verifying it.” These items were intended to capture efficacy beliefs regarding anti-fraud-related tasks rather than general internet-use competence.

To avoid an unnecessary expansion of construct boundaries, the items were restricted mainly to confidence judgments about the ability to perform anti-fraud-related tasks and did not directly assess threat awareness, behavioral frequency, value orientations, or preventive intention. The scale consisted of eight items. Standardized factor loadings ranged from 0.739 to 0.889, composite reliability was 0.949, average variance extracted was 0.698, and Cronbach’s alpha was 0.948, indicating strong measurement performance in the present sample.

#### 3.2.4 Online scam prevention behavior

Online scam prevention behavior was measured on the basis of the Online Scam Prevention Behavior scale proposed by Intarakamhang et al. (2025). This instrument was developed to assess the preventive actions individuals adopt when facing online risks and is highly consistent with the conceptual definition of the dependent variable in the present study. While preserving the original behavior-oriented measurement logic and item structure, this study made limited contextual adjustments to several items, mainly by refining the behavioral targets and specific scenarios without changing the types of behavior being measured. Representative items include “I avoid sharing personal information online” and “I avoid visiting websites that could threaten my personal information or property,” both of which directly reflect information protection and risk-avoidance behavior among Chinese university students in online scam contexts.

In the measurement evaluation, one underperforming item was removed on the basis of the criterion that standardized factor loadings should exceed 0.50 (Hair et al., 2019). After revision, standardized factor loadings ranged from 0.566 to 0.819, composite reliability was 0.921, average variance extracted was 0.542, and Cronbach’s alpha was 0.917. These results indicate good reliability and convergent validity in the present sample.

### 3.3 Data analysis

This study employed an analytical strategy that integrated structural equation modeling and latent profile analysis. First, structural equation modeling was conducted in R using the lavaan package, and model parameters were estimated with the maximum likelihood method. The reliability and validity of the measurement model were assessed through confirmatory factor analysis, including standardized factor loadings, composite reliability, and average variance extracted. Because online fraud susceptibility was conceptualized as a multidimensional construct, two supplementary analyses were conducted to examine its measurement structure and dimension-specific path patterns. First, an alternative correlated first-order-factor CFA model was estimated for the OFSS dimensions to examine whether the five susceptibility dimensions could be empirically distinguished without imposing a second-order susceptibility factor. Second, supplementary dimension-specific structural models were estimated. In each model, the second-order online fraud susceptibility construct was replaced by one first-order susceptibility dimension, namely monetary motivation, heuristic processing, financial risk preference, anti-fraud knowledge, or susceptibility to social influence, while the measurement models for threat awareness, anti-fraud self-efficacy, and online scam prevention behavior and the structural paths among these variables were retained. These analyses were used to examine the empirical distinguishability of the five susceptibility dimensions and to identify dimension-specific path patterns when each dimension was embedded in the proposed theoretical framework. Because the five dimension-specific models contained different predictor indicators, they were not used for direct fit-based comparison with the second-order model.

Discriminant validity was evaluated using the heterotrait-monotrait ratio of correlations (HTMT) criterion (Henseler et al., 2015). On this basis, a structural model was specified to estimate the theoretically proposed paths, and model fit was evaluated using chi-square divided by degrees of freedom, the comparative fit index, the Tucker-Lewis index, the root mean square error of approximation, and the standardized root mean square residual (Hu and Bentler, 1999). The lavaan package is an open-source R package designed for latent variable modeling and is widely used for path analysis, confirmatory factor analysis, and structural equation modeling (Rosseel, 2012). Indirect effects were tested using bias-corrected bootstrapping with 5,000 resamples, and significance was determined on the basis of 95% confidence intervals (Preacher and Hayes, 2008).

Beyond the variable-centered analysis, this study further used Mplus 8.3 to conduct latent profile analysis based on the five dimensions of online fraud susceptibility. One-class through four-class solutions were estimated sequentially, and the optimal number of classes was determined by jointly considering the Akaike information criterion, Bayesian information criterion, sample-size adjusted Bayesian information criterion, entropy, the Vuong-Lo–Mendell–Rubin adjusted likelihood ratio test, the bootstrap likelihood ratio test, class size, and model interpretability (Muthén and Muthén, 2017). After identifying the latent profiles, the Bolck-Croon-Hagenaars method was employed to compare differences across profiles in threat awareness, anti-fraud self-efficacy, and online scam prevention behavior. Because the Bolck-Croon-Hagenaars approach is based on the three-step framework and can account for classification error in subsequent comparisons, it is particularly suitable for comparing latent classes on continuous distal outcomes while minimizing the influence of auxiliary variables on class formation (Bolck et al., 2004; Asparouhov and Muthén, 2014; Bakk and Vermunt, 2016).

## 4 Results

### 4.1 Descriptive statistics and correlations

Before testing the hypothesized relationships, descriptive statistics and bivariate correlations were examined for the main variables. Following the original scale design, online fraud susceptibility comprises five dimensions: monetary motivation, heuristic processing, financial risk preference, anti-fraud knowledge, and susceptibility to social influence. Higher scores on each dimension indicate stronger victimization susceptibility in the corresponding aspect. The heuristic processing and anti-fraud knowledge dimensions were reverse coded and then aligned in the direction of susceptibility.

As shown in Table 2, all variables demonstrated acceptable internal consistency, with Cronbach’s alpha coefficients ranging from 0.842 to 0.948, indicating satisfactory reliability. In terms of correlations, with the exception of monetary motivation, the major dimensions of online fraud susceptibility were all significantly and negatively associated with online scam prevention behavior. Among these dimensions, heuristic processing (*r* = −0.489, *p* < 0.001), anti-fraud knowledge (*r* = −0.538, *p* < 0.001), and financial risk preference (*r* = −0.225, *p* < 0.001) showed relatively stronger associations. At the same time, both threat awareness (*r* = 0.481, *p* < 0.001) and anti-fraud self-efficacy (*r* = 0.692, *p* < 0.001) were significantly and positively associated with online scam prevention behavior. These preliminary results were broadly consistent with the proposed theoretical expectations and provided an initial basis for the subsequent model testing.

**Table 2:** Descriptive statistics, composite-score correlation matrix, and internal consistency coefficients.

Variable	M	SD	1	2	3	4	5	6	7	8	α
1. MM	3.612	0.848	—								0.842
2. HP	1.889	0.654	−0.229***	—							0.902
3. FR	1.708	1.003	0.140***	0.171***	—						0.936
4. AK	1.658	0.645	0.021	0.447***	0.240***	—					0.901
5. SS	3.018	0.992	0.284***	0.119**	0.281***	0.166***	—				0.881
6. TA	4.425	0.654	0.085*	−0.312***	−0.178***	−0.320***	0.000	—			0.931
7. SE	4.037	0.698	−0.035	−0.393***	−0.063	−0.442***	−0.130**	0.370***	—		0.948
8. PB	4.294	0.570	−0.030	−0.489***	−0.225***	−0.538***	−0.191***	0.481***	0.692***	—	0.917

MM, monetary motivation; HP, heuristic processing; FR, financial risk preference; AK, anti-fraud knowledge; SS, susceptibility to social influence; TA, threat awareness; SE, anti-fraud self-efficacy; PB, online scam prevention behavior. Pearson correlation coefficients were calculated on the basis of composite scores for each construct. *p < 0.05, **p < 0.01, ***p < 0.001.

### 4.2 Measurement model assessment

To evaluate the overall measurement model, a confirmatory factor analysis was conducted. The results indicated a good model fit, with χ^2^/df = 3.209, CFI = 0.920, TLI = 0.914, RMSEA = 0.054, and SRMR = 0.052. All fit indices met commonly recommended thresholds (Hu and Bentler, 1999), suggesting that the model adequately represented the data.

In addition to overall model fit, convergent validity was supported by the standardized factor loadings, composite reliability values, and average variance extracted. Discriminant validity was assessed using the heterotrait–monotrait ratio (HTMT). Although the Fornell–Larcker criterion is widely used for assessing discriminant validity (Fornell and Larcker, 1981), prior research has shown that it may lack sensitivity in detecting discriminant validity issues under certain conditions. Therefore, the HTMT was adopted as a more stringent criterion (Henseler et al., 2015). As shown in Table 3, all HTMT values were below the conservative threshold of 0.85, indicating satisfactory discriminant validity among the latent constructs. Taken together, these results demonstrate that the measurement model achieved acceptable standards in terms of overall fit, convergent validity, and discriminant validity, thereby providing a solid foundation for subsequent structural model analyses.

**Table 3:** Discriminant validity results based on the HTMT criterion.

Construct	1	2	3	4	5	6	7	8
1. MM	—							
2. HP	0.216	—						
3. FR	0.117	0.177	—					
4. AK	0.068	0.491	0.236	—				
5. SS	0.323	0.111	0.305	0.175	—			
6. TA	0.056	0.340	0.181	0.347	0.025	—		
7. SE	0.055	0.422	0.061	0.472	0.135	0.390	—	
8. PB	0.107	0.531	0.224	0.592	0.208	0.518	0.743	—

Entries are heterotrait-monotrait ratio (HTMT) values. All HTMT values were below the recommended threshold of 0.85, indicating satisfactory discriminant validity among the constructs. Abbreviations are the same as those reported in Table 2.

To further examine the dimensional structure of online fraud susceptibility, an alternative correlated first-order-factor CFA model was estimated for the OFSS dimensions. In this model, monetary motivation, heuristic processing, financial risk preference, anti-fraud knowledge, and susceptibility to social influence were specified as five correlated first-order latent factors without imposing a second-order susceptibility factor. The model showed acceptable fit, χ^2^ = 1194.000, df = 242, CFI = 0.929, TLI = 0.919, RMSEA = 0.072, 90% CI [0.068, 0.076], and SRMR = 0.053. The standardized factor loadings ranged from 0.632 to 0.958, and all were statistically significant. These results support the empirical distinguishability of the five susceptibility dimensions. At the same time, because the present study conceptualizes online fraud susceptibility as an overall multidimensional vulnerability structure, the second-order specification was retained for the main structural and mediation analyses as a parsimonious representation of the overall construct.

These discriminant validity results provide evidence against severe conceptual redundancy among the focal constructs and support the interpretation that the relatively high explained variance in online scam prevention behavior should be considered in relation to the multivariable theoretical model rather than attributed solely to construct overlap.

### 4.3 Common method Bias test

Because the study relied on self-reported questionnaire data collected at a single point in time from a single source, common method bias was assessed from both procedural and statistical perspectives. At the procedural level, several steps were taken during data collection to reduce this risk, including anonymous participation, careful item wording, broader sample heterogeneity, and restrictions on repeated submissions from the same IP address. At the statistical level, and following the recommendations of Podsakoff et al. (2003) and the analytic logic proposed by Williams et al. (1989, 2010), a supplementary test based on an unmeasured latent method construct was conducted.

The results showed that, in Harman’s single-factor test, the first unrotated factor accounted for 29.12% of the total variance, indicating that no dominant single-factor pattern was present. In addition, the single-factor model fit the data poorly, with χ^2^ = 18,591.59, df = 1,080, χ^2^/df = 17.21, CFI = 0.396, TLI = 0.369, RMSEA = 0.146, and SRMR = 0.141. These values were substantially worse than those of the original measurement model. Further analysis showed that, after introducing a common method factor orthogonal to all substantive constructs into the original measurement model, overall fit changed only minimally. The baseline measurement model yielded χ^2^ = 3,376.248, df = 1,052, CFI = 0.920, TLI = 0.914, RMSEA = 0.054, and SRMR = 0.051, whereas the unmeasured latent method construct model yielded χ^2^ = 3,375.481, df = 1,051, CFI = 0.920, TLI = 0.914, RMSEA = 0.054, and SRMR = 0.051. Moreover, the standardized loadings of the method factor were uniformly low, approximately 0.076 to 0.146, and none reached statistical significance. The standardized loadings of the substantive indicators also changed only marginally after the method factor was introduced.

Overall, these results suggest that no dominant common method factor was detected. Nevertheless, because all focal variables were measured using self-reported questionnaires at a single time point, residual common method variance cannot be completely ruled out, and the magnitude of the path estimates should be interpreted cautiously in light of the study design.

### 4.4 Results of structural model

**Figure 2:**
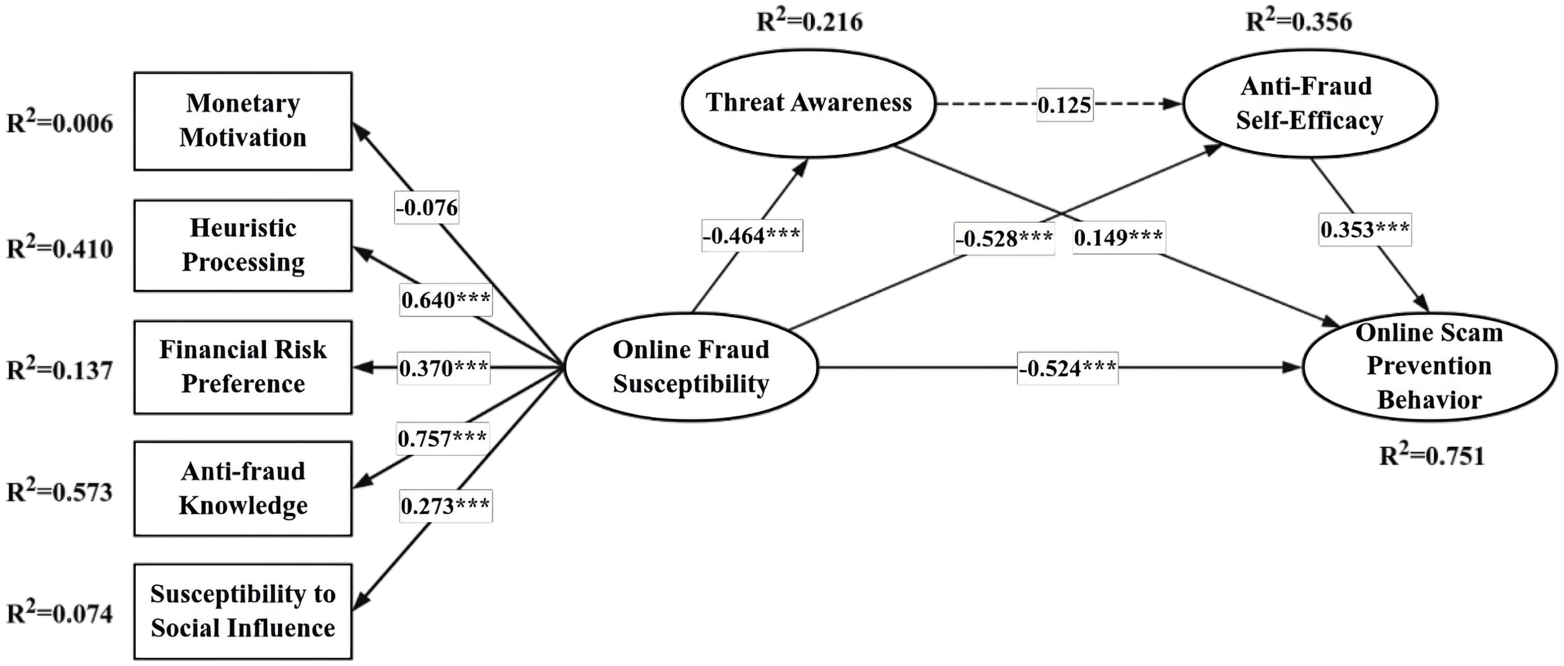
Structural model and standardized path coefficients. *p < 0.05, **p < 0.01, ***p < 0.001.

After the measurement model received satisfactory support and no dominant common method factor was detected, the structural model was estimated to test the hypothesized relationships. The model demonstrated good overall fit, with χ^2^/df = 3.352, CFI = 0.913, TLI = 0.908, RMSEA = 0.055, and SRMR = 0.070, indicating an acceptable match between the theoretical model and the sample data. As shown in Figure 2, and consistent with the measurement-model evidence reported above, online fraud susceptibility was specified as a second-order latent construct as a parsimonious representation of the overall susceptibility structure. This higher-order representation should therefore be interpreted in conjunction with the loadings of the first-order dimensions. The second-order factor was mainly reflected by heuristic processing, financial risk preference, insufficient anti-fraud knowledge, and susceptibility to social influence, whereas monetary motivation showed a weak and non-significant contribution. Therefore, the structural paths involving online fraud susceptibility should be understood as reflecting the overall effect of a multidimensional vulnerability structure rather than the effect of a fully homogeneous single trait.

In terms of structural paths, the results presented in Table 4 show that online fraud susceptibility was significantly and negatively associated with threat awareness, anti-fraud self-efficacy, and online scam prevention behavior. Both threat awareness and anti-fraud self-efficacy were significantly and positively associated with online scam prevention behavior, whereas the path from threat awareness to anti-fraud self-efficacy did not reach statistical significance.

**Table 4:** Bootstrap estimates of structural paths and indirect effects.

Hypothesized path	*β*	95% CI	*p*-value	Result
Direct effects				
FS → TA	−0.464	[−0.590, −0.339]	<0.001	Significant
FS → SE	−0.528	[−0.674, −0.383]	<0.001	Significant
TA → SE	0.125	[−0.026, 0.277]	0.105	Not significant
TA → PB	0.149	[0.035, 0.264]	0.011	Significant
SE → PB	0.353	[0.240, 0.467]	<0.001	Significant
FS → PB	−0.524	[−0.695, −0.353]	<0.001	Significant
Indirect effects				
FS → TA → PB	−0.069	[−0.123, −0.016]	0.011	Significant
FS → SE → PB	−0.187	[−0.252, −0.122]	<0.001	Significant
FS → TA → SE → PB	−0.021	[−0.046, 0.005]	0.118	Not significant
Total effects				
Total indirect effect	−0.277	[−0.378, −0.175]	<0.001	—
Direct effect	−0.524	[−0.695, −0.353]	<0.001	—
Total effect	−0.801	[−0.892, −0.709]	<0.001	—

FS, online fraud susceptibility; TA, threat awareness; SE, anti-fraud self-efficacy; PB, online scam prevention behavior; MM, monetary motivation; HP, heuristic processing; FR, financial risk preference; AK, anti-fraud knowledge; SS, susceptibility to social influence.

With respect to explanatory power, the model accounted for 21.6% of the variance in threat awareness, 35.6% of the variance in anti-fraud self-efficacy, and 75.1% of the variance in online scam prevention behavior. The R^2^ value for online scam prevention behavior was relatively high but interpretable within the present multivariable latent-variable model. First, R^2^ represents the proportion of variance jointly accounted for by all predictors in the model, rather than the explanatory contribution of any single variable. Second, the model included theoretically proximal constructs that are closely related to prevention behavior, particularly anti-fraud self-efficacy, which reflects students’ perceived capability to identify, verify, and respond to online scam situations. Within the Protection Motivation Theory framework, online fraud susceptibility, threat awareness, and anti-fraud self-efficacy correspond to vulnerability, threat appraisal, and coping appraisal components that are directly relevant to protective behavior. Therefore, the high R^2^ should be interpreted as a strong explanatory association within the specified latent-variable model rather than as evidence of causal prediction or model overfitting. As an additional robustness check, the alternative four-dimensional model excluding monetary motivation is reported in Supplementary Appendix B. The direction, significance, and overall explanatory pattern of the main paths remained substantively unchanged, suggesting that the principal findings were broadly stable under the core four-dimensional specification.

### 4.5 Mediation analysis

Bias-corrected bootstrapping with 5,000 resamples was further used to test the indirect effects, and the results are also presented in Table 4. Two indirect pathways linking online fraud susceptibility to online scam prevention behavior were statistically significant: the pathway through threat awareness and the pathway through anti-fraud self-efficacy. By contrast, the sequential indirect pathway from online fraud susceptibility to threat awareness, then to anti-fraud self-efficacy, and finally to online scam prevention behavior did not reach statistical significance. At the same time, the total indirect effect was significant, and the direct path from online fraud susceptibility to online scam prevention behavior remained significant. These findings indicate that the association between online fraud susceptibility and online scam prevention behavior was statistically accounted for primarily by two parallel indirect pathways rather than by a stable sequential process.

### 4.6 Supplementary dimension-specific structural models

Supplementary dimension-specific structural models were further estimated to examine whether the five first-order susceptibility dimensions could be incorporated into the proposed theoretical framework. All five models showed acceptable fit, with CFI ranging from 0.926 to 0.951, TLI from 0.919 to 0.946, RMSEA from 0.044 to 0.051, and SRMR from 0.043 to 0.052. The results showed that heuristic processing and insufficient anti-fraud knowledge were consistently and negatively associated with threat awareness, anti-fraud self-efficacy, and online scam prevention behavior. Financial risk preference was negatively associated with threat awareness and prevention behavior, whereas susceptibility to social influence was negatively associated with self-efficacy and prevention behavior. By contrast, monetary motivation showed only a weak positive association with threat awareness and had no significant total effect on prevention behavior. These findings suggest that the overall structural pattern of online fraud susceptibility was mainly reflected in cognitive processing, knowledge preparedness, risk preference, and social influence, whereas monetary motivation played a weaker role. Detailed results are reported in Table 5.

**Table 5:** supplementary dimension-specific structural models of the five susceptibility dimensions.

Predictor	CFI	TLI	RMSEA	SRMR	TA	SE	PB	Total effect on PB
MM	0.939	0.933	0.048	0.052	0.123**	−0.051 ns	0.007 ns	0.040 ns
HP	0.926	0.919	0.051	0.049	−0.329***	−0.335***	−0.257***	−0.556***
FR	0.951	0.946	0.044	0.045	−0.196***	−0.002 ns	−0.159***	−0.257***
AK	0.937	0.931	0.047	0.051	−0.337***	−0.372***	−0.279***	−0.588***
SS	0.944	0.939	0.046	0.043	−0.003 ns	−0.139**	−0.119***	−0.203***

Standardized coefficients are reported. Each susceptibility dimension was separately entered as an exogenous latent predictor in the proposed structural model. MM, monetary motivation; HP, heuristic processing; FR, financial risk preference; AK, anti-fraud knowledge; SS, susceptibility to social influence; TA, threat awareness; SE, anti-fraud self-efficacy; PB, online scam prevention behavior. ns, not significant. **p < 0.01, ***p < 0.001.

### 4.7 Latent profile analysis of online fraud susceptibility

#### 4.7.1 Profile identification and model selection

Building on the preceding variable-centered analysis, latent profile analysis was conducted in Mplus 8.3 using the five dimensions of online fraud susceptibility—monetary motivation, heuristic processing, financial risk preference, anti-fraud knowledge, and susceptibility to social influence—as classification indicators, in order to identify latent heterogeneity among university students (Marsh et al., 2009; Muthén and Muthén, 2017). To determine the optimal number of profiles, one-class, two-class, three-class, and four-class models were estimated in sequence, and model fit was evaluated jointly on the basis of Akaike’s information criterion, the Bayesian information criterion, the sample-size adjusted Bayesian information criterion, entropy, the Vuong-Lo–Mendell–Rubin adjusted likelihood ratio test, the bootstrap likelihood ratio test, class size, and substantive interpretability (Muthén and Muthén, 2017; Nylund et al., 2007).

As shown in Table 6, the information criteria decreased as the number of classes increased, indicating progressive improvement in model fit. Compared with the one-class model, the two-class model showed substantially lower values of Akaike’s information criterion, the Bayesian information criterion, and the sample-size adjusted Bayesian information criterion, and both the Vuong-Lo–Mendell–Rubin adjusted likelihood ratio test and the bootstrap likelihood ratio test were significant, indicating that the two-class solution fit the data better than the one-class solution. The three-class model further reduced Akaike’s information criterion, the Bayesian information criterion, and the sample-size adjusted Bayesian information criterion to 6,084.149, 6,186.226, and 6,116.367, respectively, with an entropy value of 0.841. The Vuong-Lo–Mendell–Rubin adjusted likelihood ratio test remained significant, and the bootstrap likelihood ratio test also supported the superiority of the three-class model over the two-class model. Although the four-class model further lowered the information criteria, the Vuong-Lo–Mendell–Rubin adjusted likelihood ratio test was no longer significant, and entropy declined from 0.841 to 0.803, suggesting that the additional class did not produce a more stable improvement in classification. Taking these indicators together with class size and interpretability, the three-class model was retained as the optimal latent profile solution.

**Table 6:** Fit indices for latent profile models of online fraud susceptibility.

Model	AIC	BIC	aBIC	Entropy	Smallest class (%)	VLMR-LRT p	BLRT p	Decision
1-class	7521.782	7568.181	7536.426	—	100.00	—	—	Baseline model
2-class	6464.557	6538.795	6487.988	0.908	22.22	<0.001	<0.001	Improved
3-class	6084.149	6186.226	6116.367	0.841	15.56	<0.001	<0.001	Retained
4-class	5943.137	6073.053	5984.141	0.803	15.29	0.144	<0.001	Not retained

Compared with the two-class model, the three-class model achieved a more balanced result across model fit, classification precision, and class size, and was therefore retained as the final model. Although the four-class model further reduced the information criteria, the VLMR-LRT was no longer significant and entropy declined, and thus it was not adopted.

**Figure 3:**
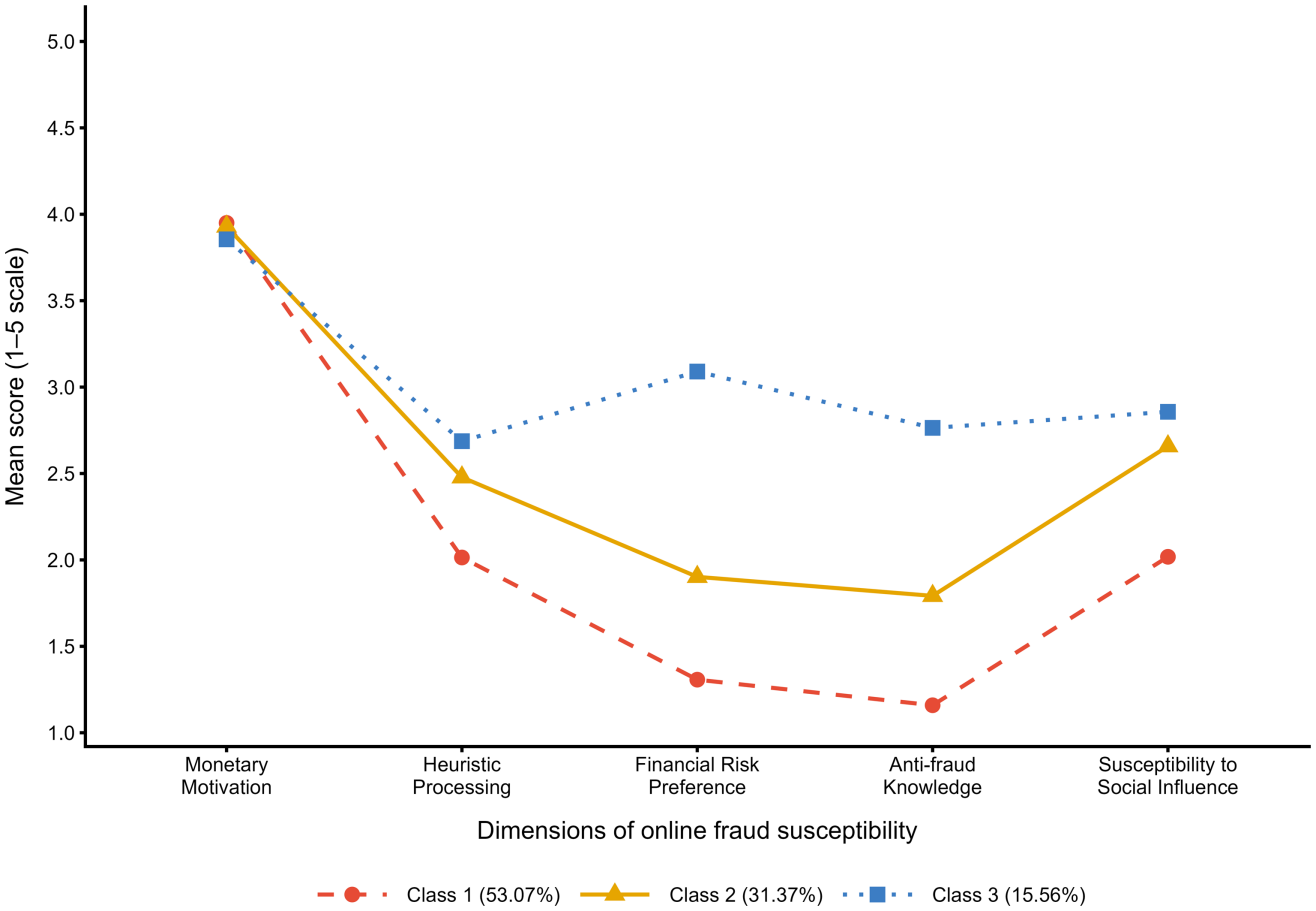
Latent profile plot of the five dimensions of online fraud susceptibility.

It should be noted that latent profile analysis focuses on latent heterogeneity reflected in the combined pattern of the five dimensions rather than on the relative loading strength of each dimension in the second-order structure (Marsh et al., 2009). For this reason, monetary motivation was retained as a classification indicator together with the other four dimensions so that the configurational characteristics of online fraud susceptibility could be represented as fully as possible. In the final three-class model, the three classes accounted for 53.07, 31.37, and 15.56% of the sample, respectively, and no class was excessively small. Based on the mean patterns shown in Table 7 and Figure 3, Class 1 showed lower levels overall on heuristic processing, financial risk preference, anti-fraud knowledge, and susceptibility to social influence; Class 3 showed higher levels overall on these dimensions; and Class 2 fell between the other two. Monetary motivation showed only limited differentiation across classes. On the basis of these overall patterns, the three profiles were labeled the low-susceptibility group, moderate-susceptibility group, and high-susceptibility group, respectively, to ensure consistency in the presentation and interpretation of the results.

**Table 7:** Means of the five dimensions across the three latent profiles of online fraud susceptibility.

Profile	Sample proportion	Monetary motivation (MM)	Heuristic processing (HP)	Financial risk preference (FR)	Anti-fraud knowledge (AK)	Susceptibility to social influence (SS)
Low-susceptibility group (Class 1)	53.07%	3.950	2.014	1.307	1.159	2.018
Moderate-susceptibility group (Class 2)	31.37%	3.929	2.478	1.902	1.792	2.658
High-susceptibility group (Class 3)	15.56%	3.855	2.687	3.090	2.764	2.857

The values shown are estimated means for the three latent profiles on the five classification indicators. For ease of interpretation and consistency with the main text, the profiles are presented according to the relabeled order: Class 1 = low susceptibility, Class 2 = moderate susceptibility, and Class 3 = high susceptibility. Higher scores on each dimension indicate stronger susceptibility in the corresponding aspect.

#### 4.7.2 Differences across profiles in outcome variables

After the three latent profiles had been identified, the Bolck-Croon-Hagenaars method was further used to compare profile differences in threat awareness, anti-fraud self-efficacy, and online scam prevention behavior. As shown in Table 8, the overall tests indicated significant differences across the three profiles for all three outcome variables.

**Table 8:** BCH comparisons of threat awareness, anti-fraud self-efficacy, and online scam prevention behavior across three latent profiles of online fraud susceptibility.

Outcome variable	Low susceptibility	Moderate susceptibility	High susceptibility	Overall test	Pairwise comparisons
Threat awareness	4.644	4.203	4.165	*χ*^2^ = 76.066, *p* < 0.001	Low > Moderate; Low > High; Moderate vs. High, ns
Anti-fraud self-efficacy	4.322	3.639	3.925	*χ*^2^ = 126.923, *p* < 0.001	Low > Moderate; Low > High; High > Moderate
Online scam prevention behavior	4.484	3.863	3.945	*χ*^2^ = 187.165, *p* < 0.001	Low > Moderate; Low > High; Moderate vs. High, ns

For ease of interpretation and consistency with Figure 3, the results are presented according to the relabeled profile order: Class 1 = low susceptibility, Class 2 = moderate susceptibility, and Class 3 = high susceptibility. The difference between the moderate-susceptibility and high-susceptibility groups was not significant for threat awareness (*p* = 0.669) or online scam prevention behavior (*p* = 0.290), but was significant for anti-fraud self-efficacy (*p* < 0.001).

More specifically, the low-susceptibility group showed higher mean scores than the other two groups on threat awareness, anti-fraud self-efficacy, and online scam prevention behavior. Pairwise comparisons further showed that, for both threat awareness and online scam prevention behavior, the low-susceptibility group scored significantly higher than the moderate-susceptibility and high-susceptibility groups, whereas the moderate-susceptibility and high-susceptibility groups did not differ significantly from one another. For anti-fraud self-efficacy, the low-susceptibility group again scored significantly higher than both of the other groups, and the high-susceptibility group also scored significantly higher than the moderate-susceptibility group. Overall, these results indicate that profile differences in online fraud susceptibility were reflected not only in the configuration of the five antecedent dimensions, but also in subsequent cognitive, efficacy-related, and behavioral outcomes.

## 5 Discussion

This study examined the factors associated with online scam prevention behavior among Chinese university students from both variable-centered and person-centered perspectives. The findings suggest that such behavior cannot be sufficiently explained by general risk awareness alone, but is better understood within a framework that integrates online fraud susceptibility, threat awareness, anti-fraud self-efficacy, and latent group differences. Compared with previous studies that have focused mainly on fraud victimization, risk exposure, or phishing-related training, the present study contributes by shifting attention to prevention behavior and by linking multidimensional susceptibility with the appraisal mechanisms of Protection Motivation Theory. It also extends prior work by showing that susceptibility-related differences operate not only through overall structural relationships, but also through distinct latent profiles among students. Building on this overall pattern, the discussion is organized around three related aspects.

### 5.1 Online fraud susceptibility and differences in online scam prevention behavior

The results indicate that online fraud susceptibility should not be treated as a static indicator of victimization risk, but rather as a susceptibility-related characteristic associated with risk judgment, capability beliefs, and prevention behavior. With the exception of monetary motivation, all major dimensions of online fraud susceptibility were significantly negatively related to online scam prevention behavior, with heuristic processing, anti-fraud knowledge, and financial risk preference showing relatively stronger associations. The second-order structure further suggests that, in this sample, online fraud susceptibility is primarily reflected in heuristic processing, financial risk preference, insufficient anti-fraud knowledge, and susceptibility to social influence.

This pattern suggests that lower levels of prevention behavior may not be primarily associated with profit-seeking tendencies, but are more closely related to limitations in cue recognition, biased risk judgment, insufficient knowledge preparation, and greater susceptibility to external influence. Such findings are broadly consistent with recent research in cybersecurity. For example, Kennison and Chan-Tin (2020) showed that knowledge, general risk-taking tendencies, and individual characteristics jointly shape risky online behavior, while Qu et al. (2024) found that fear and perceived risk of cyber fraud do not fully overlap among Chinese university students, suggesting that risk-related psychological responses are themselves structured rather than uniform. Taken together, these findings support conceptualizing online fraud susceptibility as a contextually embedded configuration of vulnerabilities, rather than a simple manifestation of monetary motivation.

The supplementary dimension-specific analyses further clarify the conceptual status of online fraud susceptibility. Although the OFSS provides a validated five-factor measurement framework, the present findings suggest that the five dimensions do not contribute equally to the structural model. Monetary motivation played a relatively weak role, whereas heuristic processing and insufficient anti-fraud knowledge showed more stable and stronger associations with threat awareness, anti-fraud self-efficacy, and online scam prevention behavior. This pattern supports interpreting online fraud susceptibility among Chinese university students as a multidimensional vulnerability structure, with cognitive processing and knowledge preparedness forming its more central components.

### 5.2 Threat awareness, anti-fraud self-efficacy, and their relative roles

The findings show that both threat awareness and anti-fraud self-efficacy are significantly associated with online scam prevention behavior, yet they do not form a stable sequential relationship. Although both variables were positively associated with prevention behavior, the path from threat awareness to self-efficacy is not statistically significant, and the sequential indirect effect is not supported. By contrast, the indirect effect through self-efficacy is stronger than that through threat awareness. This suggests that, in the context of Chinese university students, recognizing the existence of risk does not necessarily translate into confidence in one’s ability to identify, verify, and respond to potential scams.

From a theoretical perspective, this pattern does not contradict Protection Motivation Theory, but rather reflects its application in a specific context. Maddux and Rogers (1983) explicitly incorporated self-efficacy into PMT as a core cognitive component, emphasizing its role in translating cognitive appraisal into action. Empirical evidence in related domains also supports the central role of efficacy beliefs. Han et al. (2025) found that perceived threat, self-efficacy, and response efficacy all significantly influence information security behavior among Chinese university students, with efficacy-related components being more closely linked to behavioral outcomes. Similarly, Rhee et al. (2009) demonstrated that self-efficacy is closely associated with end users’ security practices, while Liang and Xue (2010) showed that threat avoidance depends not only on perceived threat but also on individuals’ confidence in their ability to implement protective actions. Arachchilage and Love (2014) further argued that knowledge and training contribute to protective behavior largely through enhancing task-specific self-efficacy. This interpretation is also consistent with recent misinformation research, in which Paciello et al. (2023) emphasized that self-efficacy in dealing with online misinformation involves specific capabilities such as evaluating the reliability of information and resisting the sharing of unverifiable content. Although misinformation and online scams are not identical phenomena, both require individuals to move beyond risk recognition and develop confidence in concrete evaluative and self-regulatory actions.

Taken together, the present findings are more consistent with a parallel relationship between threat awareness and anti-fraud self-efficacy, rather than a stable sequential process. The contribution of this study is therefore not to replace PMT’s threat-appraisal logic, but to show that, in online scam prevention, domain-specific anti-fraud self-efficacy may function as a more proximal psychological resource for preventive behavior than general threat awareness. This has direct implications for practice. Approaches that rely primarily on general risk reminders are unlikely to produce sustained behavioral change unless they are accompanied by the development of actionable skills. In this regard, Jampen et al. (2020) showed that effective anti-phishing training is typically not based on one-time warnings, but on sustained, context-embedded learning processes that are closely aligned with real-world tasks. This helps explain why self-efficacy exhibits a stronger association with prevention behavior in the present study.

### 5.3 Profile heterogeneity and implications for anti-fraud education

The person-centered analysis further suggests that differences in prevention behavior cannot be fully captured by average relationships at the sample level. The latent profiles indicate that the low-susceptibility group consistently shows higher levels of threat awareness, self-efficacy, and prevention behavior than the other groups. In contrast, the moderate- and high-susceptibility groups do not differ significantly in threat awareness or prevention behavior, but show clear differences in self-efficacy. This pattern suggests that susceptibility is not distributed along a simple continuum, but instead reflects structured heterogeneity.

This interpretation is consistent with recent person-centered research on online misinformation vulnerability. D’Errico et al. (2024) showed that adolescents’ vulnerability to racial misinformation can be organized into distinct profiles based on different configurations of cognitive, motivational, and social factors. Although the present study focuses on online scams rather than misinformation, the shared implication is that online vulnerability should not be understood only as a matter of higher or lower risk, but also as a structured configuration of underlying susceptibility characteristics. In this sense, the low-, moderate-, and high-susceptibility profiles identified in the present study provide additional evidence that online scam prevention should consider latent heterogeneity among users.

This finding is consistent with the person-oriented perspective. Bergman and Magnusson (1997) argued that variable-centered approaches are useful for identifying general relationships, but are limited in capturing typical patterns at the individual level, whereas person-centered approaches emphasize configurations of factors and the identification of common types. In this study, the value of the latent profile analysis lies in revealing distinct combinations of risk and capability underlying similar average relationships. From a practical perspective, these results suggest that uniform risk communication strategies are unlikely to meet the needs of all students. In particular, individuals with moderate susceptibility but relatively low self-efficacy may not lack awareness of risk, but rather the capacity to translate such awareness into concrete action. Jampen et al. (2020) similarly noted that effective anti-phishing interventions should account for individual differences and focus on skill development. In this sense, the person-centered findings extend the variable-centered results by highlighting the importance of differentiated, capability-oriented approaches to anti-fraud education in university settings.

At the policy level, universities could translate this approach into tiered anti-fraud education: general risk reminders for all students, and scenario-based practice in identifying suspicious information, verifying payment requests, delaying responses under pressure, protecting personal information, and using official reporting channels for students with lower self-efficacy or higher susceptibility profiles. Such differentiated training is also consistent with the person-centered implication that prevention strategies should be adapted to the specific vulnerability configurations of different student groups rather than treating fraud risk as a single undifferentiated problem. This point is also broadly consistent with recent fraud-prevention research showing that fraud risk may arise from multiple mechanisms, including pressure, opportunity, rationalization, and capability, and therefore should be addressed through mechanism-specific prevention strategies rather than through a single generalized response (Lisa et al., 2025).

## 6 Conclusion

This study investigated online scam prevention behavior among Chinese university students by examining the roles of online fraud susceptibility, threat awareness, anti-fraud self-efficacy, and latent group heterogeneity. The findings suggest that such behavior should not be understood merely in relation to perceived risk, but rather in association with the interplay among susceptibility structures, cognitive appraisals, and population-level differences. In this sense, the study extends previous work by moving from a primary focus on fraud risk and victimization toward a more prevention-oriented explanation of students’ online safety behavior.

At the theoretical level, the results support the applicability of Protection Motivation Theory while indicating that its mechanisms may operate in a more parallel manner in this context. Anti-fraud self-efficacy appears more directly associated with behavioral execution than threat awareness, which is consistent with PMT’s emphasis on self-efficacy (Maddux and Rogers, 1983) and with recent findings in the context of Chinese university students (Han et al., 2025). At the same time, online fraud susceptibility is not primarily driven by monetary motivation, but by a combination of insufficient knowledge, heuristic processing, risk preference, and susceptibility to social influence. This suggests that prevention behavior is more closely related to context-specific information processing and capability readiness than to simple opportunistic tendencies.

At the practical level, the findings point to the limitations of approaches that rely primarily on general risk reminders. While such approaches may enhance awareness, they are unlikely to produce sustained behavioral change without corresponding improvements in self-efficacy. Prior research suggests that knowledge and training may support avoidance behavior partly by strengthening self-efficacy, particularly when interventions are aligned with concrete tasks and embedded in everyday contexts (Arachchilage and Love, 2014; Jampen et al., 2020). Therefore, university-based anti-fraud education should move beyond generalized publicity toward capability-oriented and differentiated support, with greater attention to students’ practical ability to identify suspicious information, verify requests, delay decisions under pressure, protect personal information, and seek help through official channels.

Several limitations should be acknowledged. The cross-sectional and self-reported nature of the data limits causal interpretation, and residual common method variance cannot be fully excluded. Although the relatively high explained variance in online scam prevention behavior is interpretable given the use of multiple theoretically proximal predictors, it should still be treated with caution. The discriminant validity results and common method bias analyses suggest that severe construct redundancy or dominant method inflation is unlikely to fully account for the findings. The relative contributions of different susceptibility dimensions may vary across contexts. In addition, the use of convenience and snowball sampling, mainly through Anhui-based higher education networks, may limit generalizability and introduce selection bias or self-selection bias. Future research should use longitudinal, experimental, behavioral, or multi-source data and adopt stratified or probability-based sampling across different regions and institution types to further examine the stability of these relationships and refine the conceptual boundaries of online fraud susceptibility.

Overall, the findings highlight that, in the context of university students, awareness alone is insufficient. Behavioral improvement is more closely linked to the development of actionable capabilities and to the provision of targeted support based on structured differences in susceptibility.

## Data Availability

The raw data supporting the conclusions of this article will be made available by the authors, without undue reservation.
