# Supplementary material for: When awareness is not enough: online fraud susceptibility, threat awareness, anti-fraud self-efficacy and online scam prevention behavior among Chinese university students
Source: Front Psychol. 2026 Jul 7;17:1857855. doi: 10.3389/fpsyg.2026.1857855 (PMC13387045; doi:10.3389/fpsyg.2026.1857855)
Supplement: Supplementary file 1 [file Supplementary_file_1.DOCX]

**Supplementary Table S1. Measurement Instruments, Item Sources, and Construct Mapping**

**Table A1. Fraud Susceptibility Scale (OFSS)**

*(Source: Wang et al., 2025; adapted)*

| **Code** | **Administered Item (Chinese)** | **Translated Item (English)** |
| --- | --- | --- |
| MM1 | 我想有更多的钱。 | I want to have more money. |
| MM2 | 如果有更多的钱，我会比现在更加幸福快乐。 | If I had more money, I would be happier than I am now. |
| MM3 | 我羡慕那些比我有钱的人。 | I admire people who are wealthier than I am. |
| MM4 | 我不满意自己的财务状况。 | I am not satisfied with my current financial situation. |
| MM5 | 我常常觉得自己的生活费难以满足日常开销。 | I often feel that my living expenses are not enough to cover my daily spending. |
| HP6 | 我做财务相关的决定之前通常会深思熟虑。* | Before making financial decisions, I usually think carefully.* |
| HP7 | 做决定之前，我倾向于搜集一切必要的信息。* | Before making a decision, I tend to gather all the necessary information.* |
| HP8 | 做决定时，我会花时间仔细权衡利弊，分析风险与收益。* | When making decisions, I weigh the pros and cons and analyze risks.* |
| HP9 | 如果我第一次从某人或某平台上买东西，我会仔细确认对方的可信度。* | When buying from a person or platform for the first time, I verify its credibility.* |
| HP10 | 在与陌生人打交道时，在证明对方可信之前最好保持谨慎。* | When dealing with strangers, I remain cautious until their credibility is confirmed.* |
| HP11 | 对于他人告诉我的事情，我会仔细核实。* | I verify information told to me by others.* |
| FR12 | 我愿意用较多生活费参与高风险赌博或博彩活动。 | I would spend my living expenses on high-risk gambling activities. |
| FR13 | 我愿意用较多生活费购买彩票或类似博彩活动。 | I would spend my living expenses on lottery or similar gambling activities. |
| FR14 | 当与朋友打牌或娱乐时，我经常会一直玩到把身上的钱花光。 | I often keep playing until I lose all my money. |
| FR15 | 当我购买福利彩票刮刮乐时，我会很难停下来。 | I find it difficult to stop when buying lottery scratch cards. |
| FR16 | 我愿意把较多生活费用于高风险投资或投机机会。 | I would invest my living expenses in high-risk or speculative opportunities. |
| AK17 | 我知道全国反诈热线或其他官方反诈平台。* | I know the national anti-fraud hotline or official platforms.* |
| AK18 | 我下载并注册了国家反诈中心APP。* | I have downloaded and registered the anti-fraud app.* |
| AK19 | 我关注官方平台上发布的反诈宣传。* | I pay attention to anti-fraud information released on official platforms.* |
| AK20 | 我知道识别诈骗信息的基本方法。* | I know the basic methods for identifying scams.* |
| AK21 | 我了解电信网络诈骗的常见套路。* | I understand common scam tactics.* |
| SS22 | 我很难拒绝他人。 | I find it difficult to refuse others. |
| SS23 | 有人说我很容易被劝服。 | People say I am easily persuaded. |
| SS24 | 我经常担心会让他人失望。 | I often worry about disappointing others. |
| SS25 | 当别人对我的决定施加压力时，我通常会妥协。 | I usually compromise under pressure. |

**Note.** Items marked with * are reverse-coded.

**Table A2. Threat Awareness (TA) Items Used in This Study**

*(Developed for this study with reference to Intarakamhang et al., 2025, with item content aligned to the severity and vulnerability components of PMT threat appraisal)*

| **Code** | **Administered Item (Chinese)** | **Translated Item (English)** |
| --- | --- | --- |
| TA1 | 我认为如今电信网络诈骗分子经常在网络上利用他人。 | I think nowadays online scammers often exploit people online. |
| TA2 | 我认为电信网络诈骗可能对个人的生活和财产造成严重后果。 | I think online scams can have serious consequences for both life and property. |
| TA3 | 我认为电信网络诈骗相关信息可能对个体的情绪和行为产生负面影响。 | I think scam-related online content can negatively impact individuals’ emotions and behavior. |
| TA4 | 我认为电信网络诈骗可能对个人的学业、生活和财产安全造成严重不良后果。 | I think online scams can have serious adverse consequences for an individual’s studies, daily life, and financial security. |
| TA5 | 我认为在网络上粗心大意会使人更容易遭遇电信网络诈骗。 | I think being careless online makes a person more susceptible to online scams. |
| TA6 | 我认为自己在网络环境中也存在遭遇电信网络诈骗的可能性。 | I think I may also be exposed to the risk of online scams in the online environment. |

**Note.** Items were contextually adapted to capture students’ threat awareness in the online scam context. In this study, threat awareness is used as the operational expression of perceived threat in PMT. The item content mainly aligns with the perceived severity and perceived vulnerability components of PMT threat appraisal and does not assess coping ability, anti-fraud self-efficacy, behavioral frequency, or preventive action.

**Table A3. Anti-fraud Self-Efficacy Items Used in This Study**

*(Developed for this study with reference to Eastin & LaRose, 2000, and domain-specific self-efficacy logic)*

| **Code** | **Administered Item (Chinese)** | **Translated Item (English)** |
| --- | --- | --- |
| SE1 | 我有信心识别与电信网络诈骗相关的可疑信息。 | I feel confident identifying suspicious information related to online scams. |
| SE2 | 我有信心识别诈骗链接或诈骗网站。 | I feel confident identifying scam links or scam websites. |
| SE3 | 我有信心判断网络信息是否可信。 | I feel confident judging whether online information is trustworthy. |
| SE4 | 我有信心保护自己的网络个人信息。 | I feel confident protecting my personal information online. |
| SE5 | 在浏览互联网时，我有信心避免电信网络诈骗。 | I feel confident avoiding online scams while browsing the Internet. |
| SE6 | 在回复网络信息之前，我有信心进行核实。 | I feel confident verifying online information before responding. |
| SE7 | 面对可能的电信网络诈骗情境，我有信心妥善处理。 | I feel confident handling potential online scam situations. |
| SE8 | 当遇到电信网络诈骗时，我有信心寻求帮助或举报。 | I feel confident seeking help or reporting when encountering online scams. |

**Note.** Items were developed with reference to a task-based self-efficacy framework and adapted to the online scam context.

**Table A4. Online Scam Prevention Behavior Scale**

*(Source: Intarakamhang et al., 2025; adapted)*

| **Code** | **Administered Item (Chinese)** | **Translated Item (English)** |
| --- | --- | --- |
| PB1 | 我会避免与网络陌生人互动。 | I avoid interacting with online strangers. |
| PB2 | 我会屏蔽来自陌生人或潜在诈骗者的网络消息。 | I block online messages from strangers or potential scammers. |
| PB3 | 我会避免访问可能危害个人信息或财产安全的网站。 | I avoid visiting websites that could threaten my personal information or property. |
| PB4 | 我会避免在网络上透露个人信息。 | I avoid sharing personal information online. |
| PB5 | 我会为各类网络服务或账户设置高强度密码。 | I use strong passwords for all online services or accounts. |
| PB6 | 我只接受熟悉且信任的人发送的好友请求。 | I only accept friend requests from people I know and trust. |
| PB7 | 我会避免接触网络上可疑或具有诱导性的内容。 | I avoid suspicious or manipulative online content. |
| PB8 | 我会避免参与赌博、色情等可能增加受骗风险的高风险网络活动。 | I avoid engaging in high-risk online activities, such as gambling or pornography, that may increase scam risks. |
| PB9 | 我会避免与未经核实的网络陌生人进行视频通话。 | I avoid video calls with unverified online strangers. |
| PB10 | 为防止诈骗，我会及时更新手机银行或常用支付账户密码。 | To protect myself from scammers, I update my mobile banking or commonly used payment account passwords in time. |
| PB11 | 我会避免与仅在网络上认识的人线下见面。 | I avoid meeting people whom I have only met online. |
