# Supplementary material for: When awareness is not enough: online fraud susceptibility, threat awareness, anti-fraud self-efficacy and online scam prevention behavior among Chinese university students
Source: Front Psychol. 2026 Jul 7;17:1857855. doi: 10.3389/fpsyg.2026.1857855 (PMC13387045; doi:10.3389/fpsyg.2026.1857855)
Supplement: Supplementary file 2 [file Supplementary_file_2.docx]

**Appendices**

**Appendix A. Supplementary Measurement Model Results**

**Table A1. Standardized Factor Loadings, Standard Errors, Composite Reliability, and Average Variance Extracted**

| **Construct** | **Item** | **Loading** | **SE** | **p** | **CR** | **AVE** |
| --- | --- | --- | --- | --- | --- | --- |
| MM | MM1 | 0.814 | 0.037 | < 0.001 | 0.846 | 0.582 |
|  | MM2 | 0.885 | 0.035 | < 0.001 |  |  |
|  | MM3 | 0.708 | 0.043 | < 0.001 |  |  |
|  | MM4 | 0.617 | 0.045 | < 0.001 |  |  |
| HP | HP6 | 0.703 | 0.045 | < 0.001 | 0.903 | 0.609 |
|  | HP7 | 0.781 | 0.043 | < 0.001 |  |  |
|  | HP8 | 0.808 | 0.043 | < 0.001 |  |  |
|  | HP9 | 0.816 | 0.036 | < 0.001 |  |  |
|  | HP10 | 0.801 | 0.037 | < 0.001 |  |  |
|  | HP11 | 0.770 | 0.036 | < 0.001 |  |  |
| FR | FR12 | 0.637 | 0.050 | < 0.001 | 0.947 | 0.786 |
|  | FR13 | 0.901 | 0.047 | < 0.001 |  |  |
|  | FR14 | 0.942 | 0.051 | < 0.001 |  |  |
|  | FR15 | 0.951 | 0.050 | < 0.001 |  |  |
|  | FR16 | 0.958 | 0.050 | < 0.001 |  |  |
| AK | AK17 | 0.829 | 0.032 | < 0.001 | 0.903 | 0.652 |
|  | AK18 | 0.806 | 0.031 | < 0.001 |  |  |
|  | AK19 | 0.805 | 0.034 | < 0.001 |  |  |
|  | AK20 | 0.863 | 0.029 | < 0.001 |  |  |
|  | AK21 | 0.728 | 0.024 | < 0.001 |  |  |
| SS | SS22 | 0.768 | 0.038 | < 0.001 | 0.881 | 0.650 |
|  | SS23 | 0.826 | 0.034 | < 0.001 |  |  |
|  | SS24 | 0.817 | 0.037 | < 0.001 |  |  |
|  | SS25 | 0.812 | 0.036 | < 0.001 |  |  |
| TA | TA1 | 0.680 | 0.042 | < 0.001 | 0.935 | 0.706 |
|  | TA2 | 0.888 | 0.045 | < 0.001 |  |  |
|  | TA3 | 0.918 | 0.046 | < 0.001 |  |  |
|  | TA4 | 0.791 | 0.043 | < 0.001 |  |  |
|  | TA5 | 0.861 | 0.046 | < 0.001 |  |  |
|  | TA6 | 0.880 | 0.048 | < 0.001 |  |  |
| SE | SE1 | 0.871 | 0.026 | < 0.001 | 0.949 | 0.698 |
|  | SE2 | 0.889 | 0.027 | < 0.001 |  |  |
|  | SE3 | 0.881 | 0.027 | < 0.001 |  |  |
|  | SE4 | 0.833 | 0.028 | < 0.001 |  |  |
|  | SE5 | 0.854 | 0.026 | < 0.001 |  |  |
|  | SE6 | 0.791 | 0.026 | < 0.001 |  |  |
|  | SE7 | 0.817 | 0.028 | < 0.001 |  |  |
|  | SE8 | 0.740 | 0.028 | < 0.001 |  |  |
| PB | PB1 | 0.589 | 0.023 | < 0.001 | 0.921 | 0.541 |
|  | PB2 | 0.764 | 0.023 | < 0.001 |  |  |
|  | PB3 | 0.760 | 0.024 | < 0.001 |  |  |
|  | PB4 | 0.728 | 0.023 | < 0.001 |  |  |
|  | PB6 | 0.565 | 0.023 | < 0.001 |  |  |
|  | PB7 | 0.702 | 0.023 | < 0.001 |  |  |
|  | PB8 | 0.818 | 0.025 | < 0.001 |  |  |
|  | PB9 | 0.809 | 0.024 | < 0.001 |  |  |
|  | PB10 | 0.816 | 0.023 | < 0.001 |  |  |
|  | PB11 | 0.754 | 0.023 | < 0.001 |  |  |

**Note.** All factor loadings are standardized estimates. CR = composite reliability; AVE = average variance extracted. All first-order constructs exceeded the recommended thresholds for CR (> 0.70) and AVE (> 0.50), indicating satisfactory reliability and convergent validity.

**Appendix B. Robustness Check for the Alternative Four-Dimensional Fraud Susceptibility Model**

**Table B1. Measurement Model Results for the Alternative Four-Dimensional Fraud Susceptibility Model**

| **Construct** | **Item** | **Standardized loading** | **CR** | **AVE** |
| --- | --- | --- | --- | --- |
| **HP** | HP6 | 0.702 | 0.903 | 0.609 |
|  | HP7 | 0.780 |  |  |
|  | HP8 | 0.807 |  |  |
|  | HP9 | 0.816 |  |  |
|  | HP10 | 0.802 |  |  |
|  | HP11 | 0.770 |  |  |
| **FR** | FR12 | 0.636 | 0.947 | 0.786 |
|  | FR13 | 0.901 |  |  |
|  | FR14 | 0.942 |  |  |
|  | FR15 | 0.951 |  |  |
|  | FR16 | 0.958 |  |  |
| **AK** | AK17 | 0.829 | 0.903 | 0.652 |
|  | AK18 | 0.805 |  |  |
|  | AK19 | 0.805 |  |  |
|  | AK20 | 0.863 |  |  |
|  | AK21 | 0.728 |  |  |
| **SS** | SS22 | 0.768 | 0.881 | 0.650 |
|  | SS23 | 0.827 |  |  |
|  | SS24 | 0.817 |  |  |
|  | SS25 | 0.812 |  |  |
| **TA** | TA1 | 0.681 | 0.935 | 0.706 |
|  | TA2 | 0.888 |  |  |
|  | TA3 | 0.918 |  |  |
|  | TA4 | 0.791 |  |  |
|  | TA5 | 0.862 |  |  |
|  | TA6 | 0.879 |  |  |
| **SE** | SE1 | 0.871 | 0.949 | 0.698 |
|  | SE2 | 0.889 |  |  |
|  | SE3 | 0.881 |  |  |
|  | SE4 | 0.833 |  |  |
|  | SE5 | 0.854 |  |  |
|  | SE6 | 0.791 |  |  |
|  | SE7 | 0.817 |  |  |
|  | SE8 | 0.740 |  |  |
| **PB** | PB1 | 0.590 | 0.921 | 0.542 |
|  | PB2 | 0.765 |  |  |
|  | PB3 | 0.761 |  |  |
|  | PB4 | 0.729 |  |  |
|  | PB6 | 0.566 |  |  |
|  | PB7 | 0.702 |  |  |
|  | PB8 | 0.819 |  |  |
|  | PB9 | 0.810 |  |  |
|  | PB10 | 0.816 |  |  |
|  | PB11 | 0.754 |  |  |

**Note.** HP = heuristic processing; FR = financial risk preference; AK = anti-fraud knowledge; SS = susceptibility to social influence; TA = threat awareness; SE = anti-fraud self-efficacy; PB = prevention behavior. All standardized loadings exceeded 0.50. In the alternative four-dimensional model, the monetary motivation (MM) dimension was excluded, and PB5 was removed following the same treatment as in the main analysis.

**Table B2. Second-Order Factor Loadings of the Alternative Four-Dimensional Fraud Susceptibility Construct**

| **Second-order construct** | **First-order dimension** | **Standardized loading** | **R²** |
| --- | --- | --- | --- |
| **FS4** | HP | 0.680 | 0.463 |
|  | FR | 0.340 | 0.116 |
|  | AK | 0.740 | 0.547 |
|  | SS | 0.259 | 0.067 |

**Note.** FS4 = four-dimensional fraud susceptibility.

**Table B3. Structural paths and bootstrap estimates of indirect effects for the alternative four-dimensional fraud susceptibility model**

| **Hypothesized path** | **β** | **95% CI** | **p-value** | **Result** |
| --- | --- | --- | --- | --- |
| **Direct effects** |  |  |  |  |
| FS4 → TA | -0.461 | [-0.586, -0.341] | < 0.001 | Significant |
| FS4 → SE | -0.531 | [-0.678, -0.397] | < 0.001 | Significant |
| TA → SE | 0.126 | [-0.031, 0.269] | 0.103 | Not significant |
| FS4 → PB | -0.527 | [-0.698, -0.378] | < 0.001 | Significant |
| TA → PB | 0.151 | [0.031, 0.257] | 0.008 | Significant |
| SE → PB | 0.350 | [0.232, 0.451] | < 0.001 | Significant |
| **Indirect effects** |  |  |  |  |
| FS4 → TA → PB | -0.070 | [-0.122, -0.014] | 0.012 | Significant |
| FS4 → SE → PB | -0.186 | [-0.248, -0.122] | < 0.001 | Significant |
| FS4 → TA → SE → PB | -0.020 | [-0.045, 0.005] | 0.105 | Not significant |
| **Total effects** |  |  |  |  |
| Total indirect | -0.276 | [-0.366, -0.171] | < 0.001 | — |
| Total effect | -0.802 | [-0.883, -0.720] | < 0.001 | — |

**Note.** β = standardized estimate. Confidence intervals are 95% bootstrap confidence intervals based on 5,000 resamples. Under the four-dimensional fraud susceptibility specification, the direct effects of FS4 on TA, SE, and PB remain significant; the indirect effects through TA and SE remain significant; and the sequential indirect path remains non-significant, indicating that the overall mediation pattern remains primarily parallel rather than sequential.

**Table B4. Model fit indices and explained variance of the four-dimensional fraud susceptibility model**

| **Index** | **Value** |
| --- | --- |
| χ² | 3092.452 |
| df | 892 |
| χ²/df | 3.467 |
| CFI | 0.920 |
| TLI | 0.915 |
| RMSEA | 0.057 |
| SRMR | 0.058 |
| **Endogenous construct** | **R²** |
| TA | 0.212 |
| SE | 0.359 |
| PB | 0.752 |

**Note.** The model showed acceptable overall fit and explained 75.2% of the variance in PB, indicating that the alternative four-dimensional specification retained strong explanatory power.
